# Barriers to organisational resilience to climate hazards: A case study of Chikwawa, Malawi

**DOI:** 10.4102/jamba.v17i2.1750

**Published:** 2025-02-28

**Authors:** Japhet N. Khendlo, Roodheer Beeharry

**Affiliations:** 1 Faculty of Sustainable Development and Engineering, University of Mascareignes, Rose Hill, Mauritius; 2 Faculty of Environmental Sciences, Mzuzu University, Mzuzu, Malawi

**Keywords:** resilience, climate change, organisation, analytical hierarchy processing, climate change, floods, hydrometeorological hazards

## Abstract

Malawi faces severe climate change impacts, with 30 climate-related disasters recorded in 20 years, causing over 4000 deaths, affecting 2.6 million people and resulting in economic losses of over $1 billion. The southern region, especially Chikwawa District, is hit the hardest, experiencing 40% of these disasters. In light of this, the study aimed to assess organisations’ capacity and obstacles to collaborative approaches for adapting and building resilience to climate change-induced extreme weather events. Primary data were collected through a questionnaire distributed among 25 organisations, involving 325 participants. Thematic analysis was employed for qualitative data analysis, and the analytical hierarchy processing (AHP) method was applied to analyse intra-organisational challenges or obstacles to adopting climate resilience strategies. Alarmingly, 90% of organisations suspended operations because of climate-related disasters, with only 5% engaged in flood mitigation approaches. About 67% lacked flood abatement measures, and only 4% had conducted risk assessments. Most enterprises relied on government (80%) and Non-governmental organisations (NGOs) (70%) for resilience. Additionally, 85% of the organisations did not act collectively during extreme weather events, facing challenges such as lack of planning, adaptive capacity, leadership and funding. The results of this research offer a baseline for the organisations within the study area to map the way forward in making sure that the relentless impact of climate change-induced hazards should not always turn into disasters for their livelihoods and also the community at large.

**Contribution:**

This study provides a methodology for the identification of barriers to fostering a culture of proactive organisational adaptation to the escalating impacts of climate change for safeguarding lives and livelihood within a neighbourhood.

## Introduction

Worldwide, the impacts of climate change are increasing in frequency and intensity, particularly in terms of catastrophic floods, storms, droughts and heatwaves (IPCC 2023). Among meteorological disasters, flooding has been witnessed as one of the most dangerous and devastating type of weather events. Countries are witnessing an increase in the unpredictability and severity of flood hazards which often culminate into massive loss of lives and livelihoods together with damage to crucial infrastructure and property. Among the recent series of extreme flood events worldwide in 2022, we can recall the deadly floods in Pakistan, which claimed 1739 lives and submerged one-third of the country, affecting 33 million people (OCHA, 2023). The latter floods damaged most of the water systems in affected areas, forcing more than 5.4 million people to rely solely on contaminated water from ponds and wells. It also caused $14.9 billion of damage and $15.2 billion of economic losses (UNICEF 2022). The year 2022 has also witnessed fatal floods in Brazil with 57 deaths and thousands displaced and also in Australia where 23 people died and 20 000 homes and businesses were flooded (Alcantara et al. 2023; Kushwaha et al. 2024). Moreover, in July 2023, floods in India resulted in at least 100 casualties and impacted at least 1000 people (Liu et al. 2024).

African countries are also among regions increasingly experiencing destructive storms and floods including landslides as a result of climate change effects (IPCC 2022). In 2022, floods severely impacted countries like Chad, Liberia, Nigeria, Niger, Democratic Republic of Congo (DRC), Gambia, Mauritania, the Central African Republic, Guinea, Cote d’Ivoire, Senegal, Ghana, Cameroon, Mali and Burkina Faso (WHO 2023). In 2019, Mozambique was hit in a short time interval by two consecutive severe tropical cyclones, namely Idai and Kenneth, which caused loss of lives and brought huge destruction to the country. Cyclone Idai caused the deaths of 603 people and left more than 1.5 million people affected. Just 6 weeks later, cyclone Kenneth resulted in the deaths of 45 people and affected over 280 000 people. According to the UN Humanitarian Country Team, 3 months after the passage of the cyclones more than half a million people were reportedly still living in destroyed or structurally damaged homes, while another 70 000 people still remained displaced in emergency accommodations. In 2021, flooding affected 1.4 million people in 15 countries in West and Central Africa (IFRC 2021). In 2023, severe flooding devastated communities in the DRC where 460 people have been reported dead including huge losses in livelihoods, infrastructure and materials. During the same period, 131 people lost their lives because of floods in Rwanda and about 6000 homes were destroyed, roads were swept away and crops and livestock were wiped out (IDMC 2023). Between 2015 and 2022, floods killed more than 500 people in Ghana and destroyed about 60 000 houses including about 136 000 acres of farmland. The United Nations Refugee Agency has mentioned more than 3.4 million displaced people on the African continent because of floods in the year 2022 (OCHA 2022).

Malawi is among the African countries acutely impacted by the effects of climate change, with the frequency and intensity of devastating floods and cyclones increasing in recent years. In particular, the Chikwawa District has been disproportionately affected, experiencing significant and far-reaching consequences from these climate-related hazards. The latter include large number of people getting killed; extensive destruction of infrastructure; huge damage to agriculture, thereby worsening food insecurity and disruption of water and sanitation systems with acute risk of waterborne diseases such as cholera. In 2015, flooding caused the death of 54 people, and 121 000 people were hugely affected. In 2019, cyclone Idai left 60 dead and about 1 million people suffered from major losses. Homes and public infrastructure were destroyed, agriculture fields and livestock were washed away and substantial damage was caused to the sanitation facilities. In 2021, 52 people died during cyclone Kenneth, and in 2022, cyclone Ana brought about 46 deaths and left 4967 in distress. In 2023, at least 400 people lost their lives as flash floods led to multiple debris flows and mudslides during cyclone Freddy, and about 83 870 people were left afflicted (DoDMA 2023). The total damages caused by tropical cyclone Freddy in the housing sector, in the most highly impacted area, amount to $113.45 million. The total recovery cost for the physical damages and economic losses is estimated at $680.36m, and recovery interventions would span a period of 5 years (GoM 2023). However, from the observed recent trend in the occurrence of climate-related hazards in Malawi, it is quite obvious that before the completion of the recovery from the last event, another meteorological disaster may strike. Thus, there is an acute need to act quickly and manage interventions to be completed in the shortest term.

Climate change is already adversely affecting the lives of billions of people throughout the globe but the distress is much more acute in low-income nations such as Malawi. The latter country is one of the poorest countries in the world ranked at 174 out of 189 countries on the Human Development Index. The projections in the IPCC reports clearly show that climatic hazards such as floods will get worse over the coming years and decades. Thus, it is not enough to consider current levels of risk alone, and there is an acute need for building resilience for more extreme and more frequent events than we are currently witnessing (IPCC 2023). However, despite the mounting risks and relatively astronomical losses because of weather hazards, there is no evidence of much progress on adaptation measures in Malawi. The ‘Malawi 2023 Tropical Cyclone Freddy Post-Disaster Needs Assessment’ report emphasises on the importance of building back better by incorporating climate resilience into recovery efforts to reduce future disaster risks. For efficient disaster risk management (DRM) in Malawi, the latter report emphasises on the need for strengthening the organisational, technical, institutional and financing capabilities. Of the four aforementioned potentialities, the last three are quite scarce in low-income countries and may not be fully or adequately available for enhancing disaster risk resilience. Thus, there is a crucial need for maximising the organisational aspects of interventions for enhancing resilience to weather-related hazards such as floods and cyclonic conditions. Organisational capacity is also a prime requisite concerning the judicious application of scarce technical, institutional and financial resources, more importantly, in low-income countries. Thus, there is a crucial need for identifying barriers that hinder organisations in the local community, in a highly disaster-prone region like the Chikwawa District in Malawi, from becoming adequately responsive and participative to be able to contribute efficiently in the face of deadly weather-induced hazards such as floods to prevent risks from turning into disasters.

### Organisational resilience

Disaster resilience has been defined as the process by which individuals, organisations or communities gather and use their capability endowments to interact with the situation in a way that favourably adjusts and maintains functionality before, during and after the occurrence of a potential hazard. The key aspect is the capacity to anticipate, withstand, recover from and successfully adjust to adversity (Folake 2021). Thus, it is a characteristic that may be examined from an individual perspective and also from a collective or organisational standpoint (Bartuseviciene, Butkus & Schiuma 2024; Hillmann & Guenther 2021; Rodriguez-Sanchez et al. 2021). A community or society can be conceptualised as an organisation, thereby establishing a direct interconnection between organisational resilience and community resilience (Duchek 2020; Xiao & Cao 2017). Existing literature demonstrates that organisational resilience can be examined from various perspectives, including ecological, psychological and infrastructural processes within the natural disaster management framework (Klibi, Rice & Urciuoli 2018; Sahlmueller & Hellingrath 2022; Yao & Fabbe-Costes 2018). Over time, an organisation’s level of resilience evolves, with resilience often described as an intermediate state in the transition of an organisation’s status from fragility to anti-fragility. Thus, a four-level maturity model for organisational resilience (MMOR) illustrates the progression of an organisation’s capacity to adapt to adverse circumstances over time (Stocker et al. 2022). In the event of a hazard, an organisation’s status is expected to evolve through four distinct levels: fragile, robust, resilient and anti-fragile. This evolution is determined by the organisation’s ability to develop the necessary skills, traits and capacities to effectively manage disturbances in a timely and adaptive manner. If a fragile organisation is exposed to deteriorating conditions, it will inevitably collapse. In contrast, a robust organisation can adapt to predictable adverse circumstances (Bravo & Hernandez 2021; Polsky et al., 2007). However, when conditions fall outside the anticipated parameters, both fragile and robust organisations are likely to fail, as their ability to cope is limited to predefined scenarios. A resilient organisation is not merely strong but also capable of withstanding unanticipated events. At the highest level, an anti-fragile organisation goes beyond endurance, demonstrating the ability to adapt, grow and thrive under adverse conditions or during disasters (Munoz, Billsberry & Ambrosini 2022; Ruiz-Martin, López-Paredes & Wainer 2018).

According to Koronis and Ponis (2018), organisational resilience consists of three different abilities, namely: (1) the ability of an organisation to ‘bounce’ back (to survive) after a traumatic or unfavourable event; (2) the ability of an organisation to adapt to circumstances and events before they become unfavourable, traumatic or crises; and (3) the combined abilities of people to absorb crises and operationally adapt to new situations. The four major factors that contribute to organisational resilience, as identified in existing literature, include: readiness, responsiveness, adaptation and learning (Evans, Cregan & Wall 2020). Furthermore, existing research identifies four key factors influencing an organisation’s resilience: (1) leadership styles (Liu et al. 2021; Nasab & Amiri 2023); (2) dynamic capabilities (Teece, Peteraf & Leih 2016); (3) organisational learning and unlearning (Fiol & O’Connor 2017; Starbuck 2017); and (4) networks and social capital (Esen, Asik & Ege 2019).

Recurring meteorological disasters in the Chikwawa District of Malawi, with increasingly deadly outcomes, demonstrate that organisations have been unable to effectively cope with the worsening conditions. In order to foster corporate resilience, this research aimed to identify the barriers within organisations to the implementation of strategies for adaptation and enhancing resilience against devastating meteorological hazards such as floods including cyclonic conditions.

### Research aim and objectives

This study sought to identify challenges within local organisations which hindered the implementation of adaptation and resilience strategies to address the devastating impact of floods. Employing the analytical hierarchy processing (AHP) methodology, the research systematically evaluated the relative significance of various factors influencing individual organisational resilience. By assessing these factors at the organisational level and analysing sub-criteria for each, the study extracted actionable insights to guide policy makers and organisations within the study area on a path to resilient community. Ultimately, these efforts aimed to strengthen organisational resilience and promote sustainable development in the face of climate-related challenges.

The objectives of the study include the following:

- To examine the organisational-level barriers that impede collaborative initiatives aimed at enhancing resilience to climate change-induced extreme weather events.
- To evaluate the existing level of collective organisational climate resilience initiatives within the study area.
- To assess the level of awareness about the importance of collective resiliencebuilding in the face of severe floods.
- To employ AHP to identify factors affecting organisational resilience within each organisation.
- To highlight the desired collaboration needs and ideas as expressed by the representatives of the organisations themselves.

## Research methods and design

### Study area

Malawi is a landlocked country situated in Southeast Africa and is bordered by Mozambique to the south and east, Zambia to the west and Tanzania to the north. The study area in Malawi is one of the 28 districts named Chikwawa and is situated in the southern region of the country on the west bank of the Shire River. The district covers an area of 4892 km^2^ located at 106 metres above mean sea level, and it has a population of 564 684 with a population density of 117 per km^2^, whereby most people’s livelihood depends on agriculture (NSO 2018). Its geographical coordinates are 16° 46’ 0” South and 35° 17’ 0” East. The literacy rate in Chikwawa is about 58%, the electrification rate is about 6.4% and the water pipe rate is about 6.9%. Tropical weather prevails throughout the area, with distinct wet and dry seasons.

### Data collection

The research employed purposive sampling and random sampling techniques to engage with all 27 organisations within the Chikwawa District using a questionnaire that consisted of 16 open-ended questions. Purposive sampling was employed to identify all organisations within the district, while random sampling was employed to identify participants from the identified organisations. In each of the 27 organisations, between 10 and 20 respondents were chosen at random. The sample size of respondents required was verified using the Fisher’s formula in equation 1:


$$n=\frac{z^{2}(1-p)P}{e^{2}}$$ [Eqn 1]

*Z* = tabulated *z*-value (1.962), *n* = desired sample size, *P* = estimate of population percentage (0.5) and *e* = desired error allowance (±10%);

With *P* = 16 900 people, *p* = 1.962, *e* = 10%, *n* = 325; and equation 2:


$$n=\frac{1.962^{2}(1-0.5)\times 16\;900}{10^{2}}$$ [Eqn 2]

The data collected for this study consisted of audio recordings and completed questionnaires. The questionnaire was tested for reliability and validity using the Index of Item Objective Congruency (IOC) and Cronbach’s alpha values (Kamonratananun, Sujiva & Tangdhanakanond 2016; Pimdee 2021). The average IOC and Cronbach’s alpha values were found to be 0.6 and 0.76, respectively, which were all within the acceptable ranges and making the questionnaires acceptable to use for data collection. Prior to analysis, the data underwent preprocessing and cleaning. The cleaning process involved removing incomplete questions and irrelevant answers, as well as checking for response consistency (Acaps 2016; Ridzuan & Wan Zainon 2019).

Out of the initial 325 questionnaires, five were excluded from the data set after screening because of incompleteness. This resulted in a final data set of 320 respondents, representing 93% of the total data set. The audio recordings were imported into NVivo software for coding. Nodes were created to categorise the responses into relevant themes and topics. Likewise, the open-ended questions were coded by highlighting relevant text sections within the transcribed documents and assigning them to distinct nodes representing different themes.

### Thematic analysis method

Thematic analysis was conducted on the responses from the 320 eligible respondents using NVivo software. NVivo is an effective tool for transforming unstructured text into themes or codes, aiding in the classification, sorting and organisation of information. The results allowed for the categorisation of themes into two distinct classes: (1) themes conducive to enhancing collaboration at the organisational level for bolstering resilience against flood hazards and (2) themes highlighting barriers that hinder implementation of resilience against hazardous events.

### Analytic Hierarchy Process analysis method

The analytical hierarchy process, developed by Saaty (1987), is a decision-making methodology, designed to address complex problems by evaluating multiple alternatives based on a hierarchical set of criteria. Analytical hierarchy processing organises decision criteria, sub-criteria and objectives into a hierarchical structure to determine the relative importance of alternatives (Leal 2020; Russo & Camanho 2015). The study utilised AHP to identify the barriers and enablers of organisational collaboration in strengthening resilience to climate change-induced hazards. The overarching objective-enhancing resilience was linked to criteria such as organisational challenges, resource constraints and policy effectiveness, which were further developed into sub-criteria identified through thematic analysis (Ozcan & Musaoglu 2020).

The initial step in analysing the computed questionnaires involved assessing overall priorities through the geometric mean method (GMM), which integrates the perspectives of all experts involved (Dano 2022). Geometric mean method was chosen for its suitability and reliability in consolidating expert priorities, deemed preferable over other methods such as compromise voting, individual models or consensus approaches because of its effectiveness in handling reciprocal judgements (Balogun et al. 2021; Kułakowski 2020). Equation 1 was employed to compute the mean values of experts’ priorities using GMM. These aggregated values were then utilised in a pairwise comparison table to determine local priorities through AHP. Ensuring proper scaling is essential prior to conducting a pairwise comparison matrix (Table 1).

**TABLE 1:** Pairwise comparison matrix of eight criteria for the analytical hierarchy processing process.

Criteria	OC	RA	IA	IR	LE	TI	PE	ER	Criteria weight
OC	1.00	1.14	1.33	1.60	2.00	2.67	4.00	8.00	0.22
RA	0.88	1.00	1.17	1.40	1.75	2.33	3.50	7.00	0.19
IA	0.75	0.86	1.00	1.20	1.50	2.00	3.00	6.00	0.17
IR	0.63	0.71	0.83	1.00	1.25	1.67	2.50	5.00	0.13
LE	0.50	0.57	0.67	0.80	1.00	1.33	2.00	4.00	0.11
TI	0.38	0.43	0.50	0.60	0.75	1.00	1.50	3.00	0.08
PE	0.25	0.29	0.33	0.40	0.50	0.67	1.00	2.00	0.06
ER	0.13	0.14	0.17	0.20	0.25	0.33	0.50	1.00	0.03

ER, ethical and social responsibility; IA, innovation and adaptability; IR, impact on resilience; LE, level of engagement; OC, organisational capacity; PE, past expertise and experience; RA, resource availability; TI, technological integration.

The consistency index (CI), calculated using Equation (4), was used to assess the consistency of individual responses within a decision-making framework. Similarly, the consistency of weight was evaluated through the consistency ratio (CR), determined using Equation (5) (Taherdoost 2017). A CR value below 0.1 indicates that the factor weights derived from the comparison matrix are consistent. Conversely, a CR exceeding 0.1 indicates inconsistency in the comparison matrix, necessitating revision. In this study, the CR falls below 0.1 as shown further in the text, indicating consistency of the assessment in equation 3, 4 and 5:


$$\Pi =\sqrt[{n}]{a_{1}*a_{2}*a_{3}*a_{4}******a_{n}}$$ [Eqn 3]

where *n* = the number of respondents; *a* = the value scored by each respondent.

**max*λ* = 8.017870**


$$\begin{array}{l} CI=\frac{max\;\lambda -n}{n-1}\\ CI=\frac{8.01870-8}{8-1}=0.003 \end{array}$$ [Eqn 4]

$$\begin{array}{l} CR=\frac{CI}{RI}\\ CR=\frac{0.003}{1.40}=0.002<0.1 \end{array}$$ [Eqn 5]

Where *CI* is the consistency index, *max*λ is the eigenvector value, *RI* is the random index which varies based on the size of the comparison matrix.

This analysis was used to evaluate organisational performance and identify weak areas that impeded organisation resilience efforts within each organisation. The overall priority ranking of the criteria from highest: Organisational capacity: 0.22, Resource availability: 0.19, Innovation and adaptability: 0.17, Impact on resilience: 0.13, Level of engagement: 0.11, Technological integration: 0.08, Past expertise and experience: 0.06 and Ethical and social responsibility: 0.03. Subsequently, these factors were ranked to identify overarching traits within the sub-criteria of the main factors, ensuring resilience. Organisational capacity was ranked 8, resource availability 7, innovation and adaptability 6, impact on resilience 5, level of engagement 4, technological integration 3, past expertise and experience 2 and ethical and social responsibility 1.

**FIGURE 1:**
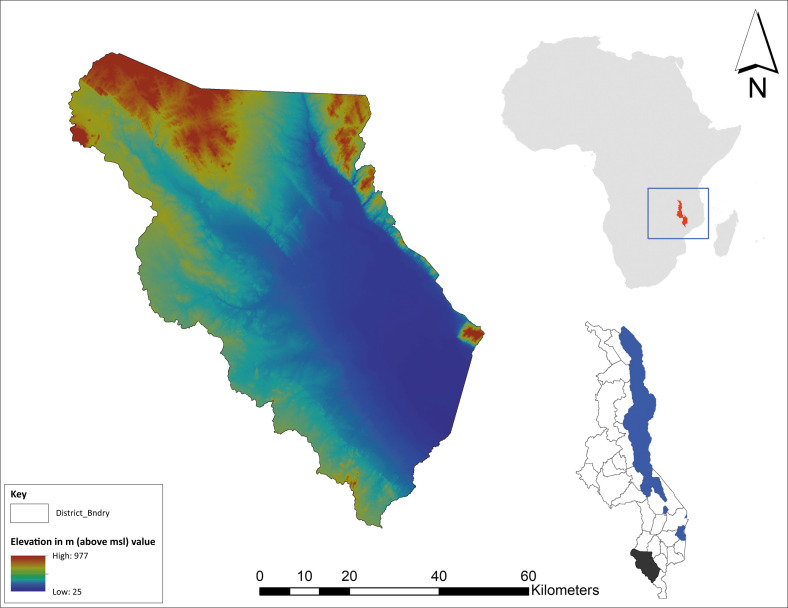
Map of Chikwawa District: the Study Area.

### Ethical consideration

Ethical clearance to conduct this study was obtained from the Universite’ Des Mascareignes Ethics committee on 15 November 2023. The researchers adhered to ethical principles in social science research by not disclosing the names and positions of participants to protect their identities, and an ethical clearance letter was obtained for the institution.

## Results

### Findings concerning thematic analysis

**FIGURE 2:**
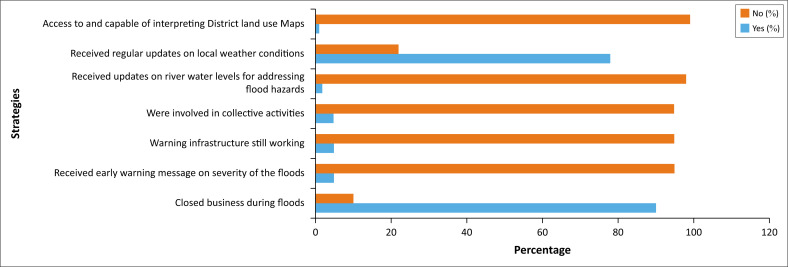
Conditions experienced by organisations during severe floods caused by cyclone Freddy in 2023.

The analysis provided insight into four key organisational-level issues regarding flood hazards: types of vulnerability, disaster preparedness, obstacles for cooperation initiatives and types of desired collaboration initiatives. This strategy allowed for a comprehensive understanding of these four issues. The constraints and challenges experienced by organisations during the recent floods triggered by cyclone Freddy in 2023 are summarised in Figure 2.

### Level of preparedness

Thematic analysis of the challenges experienced by organisations during the floods caused by cyclone Freddy last year (2023) reveals varying levels of disaster preparedness. Approximately 85% of organisations have not implemented any preparedness measures, while 10% of organisations indicate plans to relocate to less vulnerable areas. Additionally, 4% report initiating risk assessments, and only 1% are actively implementing comprehensive measures, such as constructing flood protective infrastructures, conducting drills and simulations, investing in backup power systems, training staff in emergency response protocols and establishing internal communication frameworks within their organisations.

### Factors hinder cooperative collaborations

**FIGURE 3:**
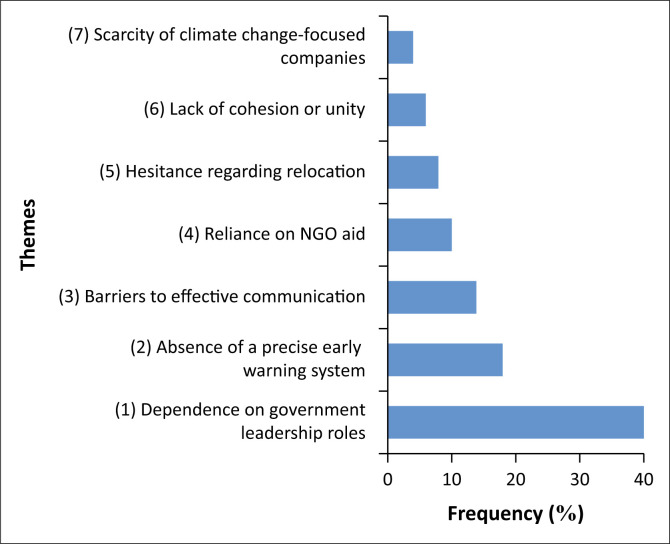
Ranking of barriers that hinder the development of resilience strategies within the study area.

The factors that may hinder cooperative collaboration within the organisations were identified through a thematic analysis conducted using NVivo software. These barriers were then ranked using the rank function in Microsoft Excel. The ranking was based on the frequency with which each theme appeared in the thematic analysis. The results of this ranking are depicted in Figure 3.

### Strategies aimed to enhance collaboration

Organisations in the study area face a number of challenges in order to collaborate on issues related to climate change resilience. Thematic analysis of the collected data identified six key strategies aimed at enhancing collaboration in addressing climate change-induced hazards. These strategies include providing training and resources to local communities to build their capacity for climate adaptation and investing in research and development to promote innovative climate adaptation technologies. Advocating for policy reforms that support climate adaptation initiatives was also highlighted as critical for creating an enabling environment for resilience efforts. Additionally, implementing green infrastructure projects such as natural buffers and sustainable drainages (avoiding farming or blocking natural drainage systems) strengthens climate resilience. Conducting collective campaigns to raise awareness and improve preparedness for climate-induced hazards further enhances collaboration. Finally, a willingness to provide financial support for developing joint climate change adaptation strategies underscores the importance of shared responsibility and resource mobilisation in fostering effective partnerships.

### Findings concerning analytical hierarchy processing analysis

The AHP analysis revealed several factors where individual organisations across various sectors are performing poorly which in turn affects their collaborative efforts. Notably, factors such as continuity planning, financial stability and funding diversity and adaptive capacity emerged as areas of concern, indicating potential weaknesses in organisational resilience and long-term planning. The results align with the literature, which highlights the fact that a failure to engage in continuous planning creates an environment where organisations struggle to adapt to change, lack preparedness for crises and miss opportunities for improvement (Asare-Kyire et al. 2023; Carmeli & Dothan 2017; Pertheban et al. 2023).

**FIGURE 4:**
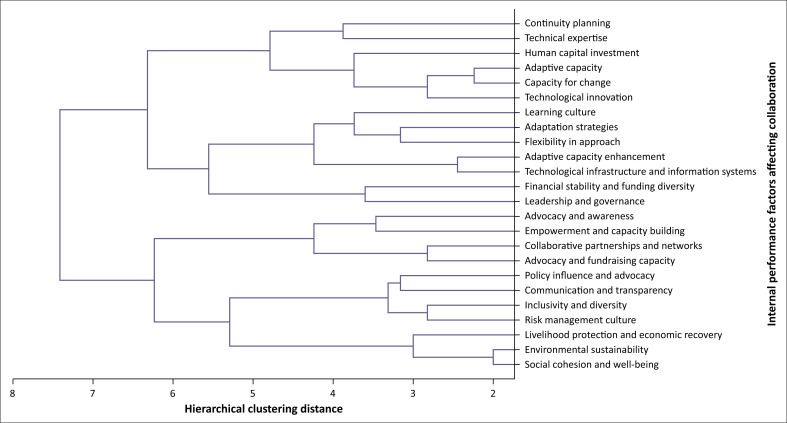
Dendrogram for poorly performing factors within organisations.

Additionally, factors such as technological infrastructure and information systems, along with collaborative partnerships and networks, underscored challenges in effectively leveraging technology and fostering strategic alliances to enhance organisational resilience. Existing literature indicates that leveraging technological advancements and networks in organisations’ operations enhances flexibility in communication, risk assessment and management. (Camarinha-Matos et al. 2019; Dubey et al. 2024; Garrido, Martín-Rojas & García-Morales 2024). The identification of these factors underscores the need for targeted interventions and resource allocation to address gaps and improve overall organisational capacity and performance. The results, as shown in Figure 4, provide valuable insights into the areas where organisations are struggling or underperforming across multiple sectors (Bourland-Davis & Graham 2017; Hafeez et al. 2024; Rasheed, Liu & Ali 2024).

Factors related to continuity planning, and learning and unlearning culture reveal potential vulnerabilities in organisational preparedness for unexpected disruptions or crises, indicating the necessity for enhanced risk- management strategies and diversified implementation of a learning and unlearning culture. Georgescu et al. (2024) highlighted the fact that an organisation’s ability to recognise and assimilate new knowledge plays a crucial role in fostering collaboration. This capacity, combined with a critical analysis of the evolving nature of operations, significantly enhances organisations’ effectiveness (Manju Prem et al. 2024; Naveed et al. 2022). Additionally, the emphasis on environmental sustainability underscores the increasing importance of addressing environmental concerns and integrating sustainable practices into organisational operations (Linnenluecke, Griffiths & Mumby 2015).

Moreover, the identification of factors such as technological infrastructure and collaborative partnerships highlights the significance of embracing digitalisation and fostering synergistic relationships with stakeholders to drive innovation and enhance organisational resilience. A study by Adomako and Nguyen (2024) found that digitalisation within organisations positively influences inter-organisational collaboration. This enhanced collaboration facilitates effective technological transfer, which in turn significantly boosts the intensity of commercialisation and cooperation efforts (Naveed et al. 2022).

In Figure 3, the dendrogram illustrates the hierarchical nature of the themes identified as areas where organisations are performing poorly internally, thereby hindering their ability to collaborate effectively with others. The dendrogram’s structure visually represents how these internal performance issues are organised from significant to the least impactful in terms of their influence on collaboration (Boyko & Tkachyk 2023; Contreras & Murtagh 2015).

The hierarchical arrangements highlight how the internal barriers of organisations are interconnected and progressively affect the capacity to engage in collaborative efforts. The length of the branches indicates the degree of dissimilarly or distance between these internal issues, helping to visualise the relative importance and clustering of the various barriers to collaboration (Lazarević & Mosurović Ružičić 2023; Dosi, Marengo & Virgillito 2021).

## Discussion

The results of the study show that almost all the organisations in the study area are vulnerable to climate change-induced extreme weather events at varying susceptible levels and lack proper strategies to work collectively to address the latter issues exacerbating the adverse impacts of climate change-induced extremes. This is evidenced by the relatively very high proportion of organisations (90%) which had to stop operations during the floods because their infrastructures became unusable during flood events of 2023. As noted from the responses of the interviewees, the disruption was not limited to physical damage but also extended to the organisation’s ability to function even remotely. Sarkar et al. (2023) and Alibašić (2024) highlighted that organisational resilience is a function of adaptive capacity and infrastructure quality which enables the continuation with minimal disruptions. The magnitude of infrastructure collapse during the recent floods underscored the urgent need for not only improving the quality of infrastructure but also ensuring comprehensive and effective urban planning.

It has been highlighted that a significant lack of knowledge exists regarding collective management, including role assignment, within organisations willing to contribute financially or materially towards shared responsibilities for building resilience against extreme flood events. Additionally, the lack of role assignment within organisations, despite their willingness to contribute to resilience building, leads to excessive reliance on government and NGO actors, resulting in ineffective, short-term solutions that fail to address the root of organisations’ vulnerability. The findings are consistent with those of Kefalas (2017) and Gazdeliani (2024), who noted that the absence of clearly defined role assignments among organisations collaborating to strengthen community resilience against extreme weather events fosters a tendency towards blame-shifting. This lack of coordination perpetuates a cycle of disaster, recovery and repetition, with increasingly devastating impacts each time a disaster strikes, as no organisation is fully aware of its responsibilities in ensuring resilience to climate change.

Despite some limited collective resilience initiatives among the organisations, they are often underdeveloped hindered by poor coordination, unclear ownership and inadequate monitoring, leaving both organisations and communities vulnerable to future flood events. Literature (Auliagisni, Wilkinson & Elkharboutly 2022; Rosmadi et al. 2023; Shah et al. 2023) highlights the fact that the absence of effective monitoring and poor coordination in resilience or adaptive efforts often result in several critical challenges. These include the inability to identify weaknesses, limited capacity to adapt to evolving conditions and misallocations of resources. Moreover, such deficiencies foster a reactive rather than proactive approach to disaster management, undermining the effectiveness of preparedness and response strategies. The lack of coordination also creates fragmented efforts and information silos, where vital information is not effectively shared among stakeholders (Bento, Tagliabue & Lorenzo 2020; De Waal et al. 2019). At the core of organisational resilience lies the capacity for organisational adaptations, as well as ongoing monitoring and evaluation of identified strategies (Inderberg 2015; Nair, Manohar & Mittal 2024; Sethi, Sushil & Gupta 2024). The absence of these measures, along with a lack of clear ownership of the identified strategies, renders the organisations and the communities at large vulnerable to the potential impact of future floods (Endendijk et al. 2024; John 2020). As the devastating impact of floods, worsened by climate change, continues to affect communities, organisational resilience must be addressed proactively and with urgency. This approach is crucial to minimising the negative consequences of extreme weather events (Chigudu & Chigudu 2015; Georgiou & Arenas 2023; Roberts 2023).

The identified obstacles for improving collaborative efforts to address climate change impacts highlight the need for a more efficient approach that fosters enhanced collaboration, particularly through clear ownership of responsibilities, in addressing localised susceptibility and vulnerability to climate change hazards (Adaptation Fund 2024; UNDRR 2020).

## Conclusion

Organisations should reframe their perspective on climate change-induced disasters, shifting from viewing them as insurmountable challenges to approaching them positively and collaboratively. This paradigm shift will empower communities to adopt adaptive strategies, improve preparedness and contribute to building long-term resilience in the face of the ongoing climate crisis. This recommendation aligns with the findings of Ahmad and Abu Talib (2015) and Ibrahim (2024), who emphasised that involving local institutions and implementing decentralisation strategies in addressing community challenges significantly enhance the sense of ownership and responsibility within communities. Measured against the four-level MMOR proposed by Ruiz-Martin et al. (2018), it is evident that the community remains vulnerable to the impacts of extreme weather events driven by climate change. This underscores the urgent need for ownership and the implementation of adaptive strategies to enhance resilience and reduce vulnerability to such events. By addressing these areas of weakness, organisations can enhance their adaptive capacity, actively contribute to collaborative efforts in dynamic environments, mitigate risks and strengthen community resilience. The results of this research provide a foundational framework for organisations within the study area and beyond to chart a path forward, ensuring that the relentless impacts of climate change-induced hazards no longer escalate into disasters, thereby promoting the effective safeguarding of communities at large.

### Study limitation

Because of limited time and lack of human resources for the survey, we could only engage the statistical minimum number of stakeholders. A larger number of participating organisations could have revealed other latent issues at organisational levels.

### Large language model statement

Some large language model (LLM) tools have been used in some cases for paraphrasing and grammatical error corrections.
